# Proliferation and differentiation of adipose tissue in prolonged lean and obese critically ill patients

**DOI:** 10.1186/s40635-017-0128-3

**Published:** 2017-03-16

**Authors:** Chloë Goossens, Sarah Vander Perre, Greet Van den Berghe, Lies Langouche

**Affiliations:** Clinical Division and Laboratory of Intensive Care Medicine, Department of Cellular and Molecular Medicine, KU Leuven, 3000 Leuven, Belgium

**Keywords:** Critical illness, Obesity, Adipogenesis, Eicosanoids

## Abstract

**Background:**

In prolonged non-obese critically ill patients, preservation of adipose tissue is prioritized over that of the skeletal muscle and coincides with increased adipogenesis. However, we recently demonstrated that in obese critically ill mice, this priority was switched. In the obese, the use of abundantly available adipose tissue-derived energy substrates was preferred and counteracted muscle wasting. These observations suggest that different processes are ongoing in adipose tissue of lean vs. overweight/obese critically ill patients.

**Methods:**

We hypothesize that to preserve adipose tissue mass during critical illness, adipogenesis is increased in prolonged lean critically ill patients, but not in overweight/obese critically ill patients, who enter the ICU with excess adipose tissue. To test this, we studied markers of adipogenesis in subcutaneous and visceral biopsies of matched lean (*n* = 24) and overweight/obese (*n* = 24) prolonged critically ill patients. Secondly, to further unravel the underlying mechanism of critical illness-induced adipogenesis, local production of eicosanoid PPARγ agonists was explored, as well as the adipogenic potential of serum from matched lean (*n* = 20) and overweight/obese (*n* = 20) critically ill patients.

**Results:**

The number of small adipocytes, PPARγ protein, and *CEBPB* expression were equally upregulated (*p* ≤ 0.05) in subcutaneous and visceral adipose tissue biopsies of lean and overweight/obese prolonged critically ill patients. Gene expression of key enzymes involved in eicosanoid production was reduced (*COX1*, *HPGDS*, *LPGDS*, *ALOX15*, all *p* ≤ 0.05) or unaltered (*COX2*, *ALOX5*) during critical illness, irrespective of obesity. Gene expression of *PLA2G2A* and *ALOX15B* was upregulated in lean and overweight/obese patients (*p* ≤ 0.05), whereas their end products, the PPARγ-activating metabolites 15s-HETE and 9-HODE, were not increased in the adipose tissue. In vitro, serum of lean and overweight/obese prolonged critically ill patients equally stimulated adipocyte proliferation (*p* ≤ 0.05) and differentiation (lipid accumulation, *DLK1*, and *CEBPB* expression, *p* ≤ 0.05).

**Conclusions:**

Contrary to what was hypothesized, adipogenesis increased independently of initial BMI in prolonged critically ill patients. Not the production of local eicosanoid PPARγ agonists but circulating adipogenic factors seem to be involved in critical illness-induced adipogenesis. Importantly, our findings suggest that abundantly available energy substrates from the adipose tissue, rather than excess adipocytes, can play a beneficial role during critical illness.

## Background

Critical illness induces a hypercatabolic response with severe wasting of lean tissue [1, 2]. Remarkably, in non-obese patients, critical illness prioritizes the maintenance of adipose tissue over skeletal muscle tissue [3]. Such preservation of adipose tissue mass during prolonged critical illness coincided with increased adipogenesis, as has been observed in subcutaneous and visceral adipose tissue biopsies of prolonged critically ill patients [4, 5]. Recently, we have shown in an animal and patient study that premorbid obesity protected against muscle wasting and weakness. Obese critically ill mice lost relatively more adipose tissue mass than lean mice but ultimately retained more adipose tissue [6]. These observations suggest that in overweight/obese critically ill patients, preservation of the adipose tissue is not prioritized but that the stored lipids in the adipose tissue are being used, which provokes a muscle-sparing effect [6]. Overall, these findings indicate an essential role for the adipose tissue during critical illness but also suggest that different processes are ongoing in lean vs. overweight/obese critically ill patients. As it appears that having abundantly available adipose tissue is beneficial during critical illness, an increase in adipogenesis could be interpreted as an attempt to ensure sufficient adipose tissue during critical illness. However, as the muscle-sparing effect coincided with a decrease in adipose tissue mass, one could also interpret an increase in adipogenesis as an undesirable preservation of adipose tissue over the skeletal muscle. We therefore hypothesize that, to preserve adipose tissue mass during critical illness, adipogenesis is increased in prolonged lean critically ill patients, but not in overweight/obese critically ill patients, who enter the intensive care unit (ICU) with excess adipose tissue.

It is currently unclear how adipogenesis is upregulated during critical illness. It was demonstrated that the nuclear receptor peroxisome proliferator-activated receptor gamma (PPARγ), the key regulator of adipogenesis, was upregulated in adipose tissue biopsies of prolonged critically ill patients and rodents [5, 7]. Of note, adipogenesis coincided with an increased accumulation of alternatively activated M2 macrophages [5, 7]. These anti-inflammatory M2 macrophages are implicated in immunity, inflammation, allergy, parasitic infections, wound healing, metabolic functions, and malignancy [8]. Although mechanisms regulating adipose tissue plasticity during health or disease are still poorly understood [9], alternatively activated M2 macrophages have been shown to produce endogenous fatty acid-derived PPARγ ligands, thereby providing local adipogenic signals and stimulating adipogenesis [10].

The first aim of this study was to investigate whether adipogenesis is differentially increased in lean and overweight/obese prolonged critically ill patients. To test this, we studied markers of adipogenesis in subcutaneous and visceral adipose tissue biopsies of lean and overweight/obese critically ill patients that were matched for demographics and for type and severity of illness. Secondly, to further unravel the underlying mechanism of critical illness-induced adipogenesis, the local production of eicosanoid PPARγ agonists was explored. Additionally, in an in vitro study, we investigated the adipogenic potential of serum from matched lean and overweight/obese prolonged critically ill patients.

## Methods

### Collection of human adipose tissue biopsies

The study protocol of the human studies had been approved by the Institutional Review Board of the KU Leuven (ML1820, ML2707, ML1094). Written informed consent was obtained from the patients’ closest family member and from healthy volunteers. ICU patients whose biopsies were collected had been included in two randomized controlled trials on the effect of intensive vs. conventional insulin therapy in the medical and surgical ICU [11, 12]. Subcutaneous adipose tissue and visceral adipose tissue biopsies from prolonged critically ill patients were harvested immediately postmortem, within minutes after death. From 85 available biopsies, we selected 24 lean patients (body mass index (BMI) ≤25 kg/m^2^) and 24 overweight/obese patients (BMI >25 kg/m^2^) who were propensity score matched on gender, age, malignancy, diabetes, APACHE II score on admission, and randomization to intensive or conventional insulin therapy (Table 1). For each patient, BMI was calculated based on height and weight data available in the patient file. As healthy references, subcutaneous adipose tissue and visceral adipose tissue biopsies (*n* = 20) were available from non-critically ill individuals with similar demographics (Table 1). Subcutaneous and visceral adipose tissue biopsies were taken intraoperatively from patients who were not critically ill and underwent elective surgery for restorative rectal resection. Biopsies were used for RNA analyses, protein analyses, and histological analyses.

**Table 1:** Baseline and outcome characteristics of matched critically ill patients of whom postmortem subcutaneous and visceral adipose tissue biopsies were studied

	Lean critically ill (*N* = 24)	Overweight/obese critically ill (*N* = 24)	*p* value^d^	Healthy reference (*N* = 20)	*p* value^e^
Baseline characteristics					
Gender (*n*, % male)	17 (71)	14 (58)	0.5	14 (70)	0.7
Age, years (mean ± SEM)	68.4 ± 2.8	68.8 ± 2.8	0.5	70 ± 2.6	0.6
BMI, kg/m^2^ (mean ± SEM)	22.5 ± 0.4	27.7 ± 0.3	**<**0.0001	24.9 ± 2.6	0.8
Diabetes mellitus (*n*, %)	4 (17)	3 (12)	1	3 (15)	1
Malignancy (*n*, %)	9 (37)	9 (37)	0.2		
APACHE II score (mean ± SEM)	25 ± 2	26 ± 2	0.5		
Diagnostic admission category (*n*)			0.3		
Cardiac surgery	1	1			
Complicated surgery^a^	1	5			
Multiple trauma or burns	0	1			
Hematologic or oncologic	4	5			
Respiratory	12	7			
Other disease exacerbations^b^	4	4			
Other sepsis	2	1			
Randomization to intensive insulin therapy (*n*, %)	10 (42)	11 (46)	1		
Received steroid therapy (*n*, %)	17 (73)	18 (85)	0.4		
Outcome					
ICU stay (median (IQR))^c^	10 (7-20)	10 (6-17)	0.2		
Cause of death (*n*)			0.5		
Cardiac/hypovolemic shock	4	4			
Multiple organ failure	8	12			
Respiratory failure	8	7			
Septic shock/therapy resistance	3	1			
Severe brain damage	1	0			

*SEM* standard error of the mean, *BMI* body mass index, *IQR* interquartile range, *ICU* intensive care unit

^a^Complicated surgery indicates patients suffering from complications after abdominal or pelvic surgery, pulmonary or esophageal surgery, or vascular surgery

^b^Cardiovascular disease, gastroenterologic or hepatic disease, neurologic disease

^c^Postmortem biopsies were collected minutes after death in the ICU. ICU stay thus reflects day of biopsy

^d^Comparison between lean and overweight/obese critically ill patients

^e^Comparison between critically ill patients and healthy references

### Cell culture study

Serum samples were collected from prolonged critically ill patients (median ICU stay at day of serum sampling was 11 days) and healthy volunteers after informed consent. The study protocol had been approved by the Institutional Review Board of the KU Leuven (ML8850). Human adipose-derived stem cells (hADSCs; Invitrogen, Ghent, Belgium) were seeded on glass coverslips (Menzel-Gläzer, Braunschweig, Germany) coated with 5% gelatin (Sigma-Aldrich, Saint Louis, MI, USA) in Roswell Park Memorial Institute (RPMI)-1640 medium (Invitrogen) containing 10% fetal bovine serum (FBS, Invitrogen) and 1% Antibiotic-Antimycotic (A/A, Invitrogen). Cells were kept in a humidified incubator at 37 °C and 5% CO_2_. After 24 h, the medium was replaced with RPMI-1640 containing 1% A/A and 10% human serum from established serum pools. Serum pools were composed of serum from either healthy controls (*n* = 47), lean prolonged critically ill patients (BMI ≤25 kg/m^2^, *n* = 20), or overweight/obese prolonged critically ill patients (BMI >25 kg/m^2^, *n* = 20). Patients and healthy volunteers were matched for age and gender. Additionally, patients were also matched for severity of illness (Table 2). After 1, 2, 3, and 4 days, cells were fixed in 6% paraformaldehyde for histological staining and messenger RNA was isolated. All serum conditions were performed in triplicate.

**Table 2:** Baseline and outcome characteristics of matched critically ill patients of whom serum samples were used in the in vitro study

	Lean critically ill (*N* = 20)	Overweight/obese critically ill (*N* = 20)	*p* value^c^	Healthy reference (*N* = 47)	*p* value^d^
Baseline characteristics					
Gender (*n*, % male)	13 (65)	12 (60)	0.9	30 (64)	1
BMI, kg/m^2^ (mean ± SEM)	22.4 ± 0.4	29.3 ± 0.9	<0.0001	24.8 ± 3.6	0.2
Age, years (mean ± SEM)	57.2 ± 3.8	56.9 ± 3.4	0.4	58 ± 2.4	0.7
APACHE II score (mean ± SEM)	18.6 ± 6	19.3 ± 6.3	0.6		
Diagnostic admission category (*n*)	2	3	0.6		
Cardiac surgery	2	1			
Complicated surgery^a^	3	5			
Multiple trauma or burns	3	3			
Hematologic or oncologic	4	1			
Respiratory	4	6			
Other disease exacerbations^b^	2	1			
Other sepsis					
Received steroid therapy (*n*, %)	0 (0)	0 (0)	1		

*SEM* standard error of the mean, *BMI* body mass index

^a^Complicated surgery indicates patients suffering from complications after abdominal or pelvic surgery, pulmonary or esophageal surgery, or vascular surgery

^b^Cardiovascular disease, gastroenterologic or hepatic disease, neurologic disease

^c^Comparison between lean and overweight/obese critically ill patients

^d^Comparison between critically ill patients and healthy references

### Gene expression and protein expression analyses

Messenger RNA was isolated, reverse transcribed, and analyzed in real time with the StepOne Plus (Applied Biosystems, Carlsbad, CA, USA) and run in duplicate. The comparative Ct method was used to analyze data. Data are expressed normalized to either RNA, 18S ribosomal 5 (*RNA18S5*) or glyceraldehyde-3-phosphate dehydrogenase (*GAPDH*) gene expression and as a fold change of the mean of the controls. Gene expression assays from Applied Biosystems were used to detect expression levels of *RNA18S5*, *GAPDH*, CCAAT/enhancer-binding protein beta (*CEBPB*), delta-like 1 homolog (*DLK1*), cyclooxygenase 1 (*COX1*), cyclooxygenase 2 (*COX2*), prostaglandin D2 synthase (*LPGDS*), hematopoietic prostaglandin D synthase (*HPGDS*), phospholipase A2 group IIA (*PLA2G2A*), arachidonate 5-lipoxygenase (*ALOX5*), arachidonate 15-lipoxygenase (*ALOX15*), and arachidonate 15-lipoxygenase type B (*ALOX15B*). PPARγ protein levels were quantified by Western blot as described previously [5]. Data are expressed relative to the means of the controls.

15-Hydroxyeicosatetraenoic acid (15s-HETE) and 9-hydroxyoctadecadienoic acid (9-HODE) concentrations were quantified in human subcutaneous and visceral adipose tissue biopsies. Lipids were extracted in methyl formate (Sigma-Aldrich) from an equal amount of protein using C18 reverse phase columns (Bond Elut C18, Agilent Technologies, Santa Clara, CA, USA). Lipid extracts were evaporated under a stream of nitrogen and reconstituted in assay buffer before 15s-HETE and 9s-HODE determination by enzyme-linked immunosorbent assays (ELISAs; 15(S)-HETE ELISA Kit, Enzo Life Sciences, Farmingdale, NY, USA; 9(+)-HODE EIA Kit, Oxford Biomedical Research, Rochester Hills, MI, USA).

### Histological staining and analyses

Subcutaneous and visceral adipose tissue was fixed in 4% paraformaldehyde and embedded in paraffin. Sections of 5 μm were stained with hematoxylin and eosin. Adipocyte cell size was measured on digital microscopy images using Adobe Photoshop CS2 (Adobe Systems, San Jose, CA, USA) and Image Processing Tool Kit (Reindeer Graphics, Asheville, NC, USA) as described previously [4]. Fixed hADSCs were stained with Oil Red O and counterstained with hematoxylin. Visualization of the cells was performed with a Zeiss AxioVert 200M microscope (Carl Zeiss, Oberkochen, Germany) equipped with an AxioCam MRC5 camera. MosaiX pictures were taken at ×5 optical zoom using Axiovision Rel 4.8 software (Carl Zeiss). Cell counting and quantification of the Oil Red O staining were performed using ImageJ software (National Institutes of Health, Bethesda, MD, USA).

### Statistics

Data are presented as box plots with median, interquartile ranges, and 10th and 90th percentiles or as bars or graphs with whiskers, representing means, and standard error of the mean (SEM). Normally distributed data were analyzed with factorial one-way analysis of variance (ANOVA) with post hoc Fisher’s LSD test for multiple comparisons. Not-normally distributed data were analyzed with parametric tests after log or (double) square root transformation if this resulted in a normal distribution; otherwise, non-parametric Mann-Whitney *U* test were used. Comparison of matched patients and healthy references was performed with Student’s *t* tests and Fisher’s exact tests for proportions. Analyses were performed using JMP 8.0.1 (SAS Institute, Tervuren, Belgium) or SPSS 22 (IBM, Brussels, Belgium). Two-sided *p* values ≤0.05 were considered statistically significant.

## Results

### Markers of adipogenesis in subcutaneous and visceral adipose tissue biopsies

**Fig. 1:**
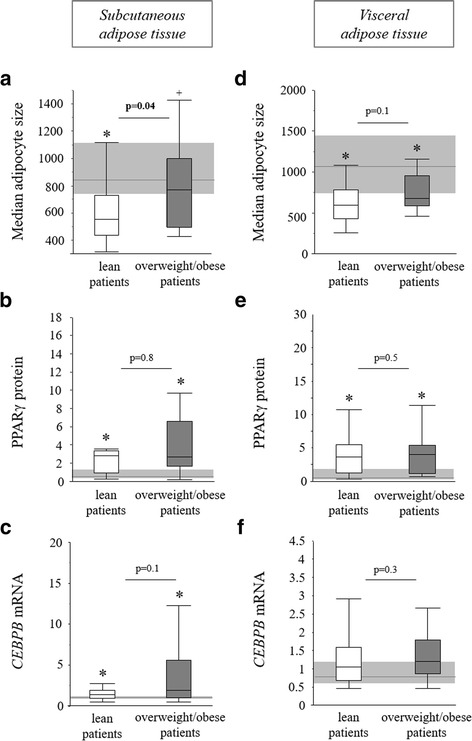
Markers of adipogenesis in lean and overweight/obese prolonged critically ill patients. Median adipocyte size in subcutaneous (**a**) and visceral (**d**) adipose tissue. Relative PPARγ protein level in subcutaneous (**b**) and visceral (**e**) adipose tissue, as detected with Western blot. Relative mRNA expression of *CEBPB* in subcutaneous (**c**) and visceral (**f**) adipose tissue. Protein levels are presented as fold change of the mean of healthy controls. Gene expression data are expressed normalized to *RNA18S5* or *GAPDH* and as a fold change of the mean of the healthy controls. *Light gray bar* median and interquartile ranges of healthy controls (*n* = 20), *white* lean prolonged critically ill patients (BMI ≤25 kg/m^2^; *n* = 24), *dark gray* overweight/obese prolonged critically ill patients (BMI >25 kg/m^2^; *n* = 24) [*p ≤ 0.05 compared to healthy controls, ^+^ *p* = 0.08 compared to healthy controls]

Compared to the subcutaneous adipose tissue of healthy controls, that of both lean and overweight/obese critically ill patients showed a lower median adipocyte size, indicative of an increased amount of small adipocytes (Fig. 1a). Furthermore, lean patients displayed a lower adipocyte size than overweight/obese patients. Corresponding with the increase in small adipocytes, PPARγ protein concentration was significantly increased in both lean and overweight/obese patients (Fig. 1b). Also, subcutaneous adipose tissue gene expression of adipogenesis-driver *CEBPB* was elevated in lean and in overweight/obese patients (Fig. 1c). Pre-adipocyte marker *DLK1* was not different between groups (*p* = 0.7, data not shown).

The visceral fat depot of both lean and overweight/obese patients showed an increased amount of small adipocytes compared to non-critically ill individuals (Fig. 1d). Also, in the visceral adipose tissue, PPARγ protein concentration was elevated in both lean and overweight/obese patients compared to controls (Fig. 1e). Visceral adipose tissue *CEBPB* gene expression (*p* = 0.1, Fig. 1f) and *DLK1* gene expression (*p* = 0.9, data not shown) were not different between groups.

Patients on intensive insulin therapy or on steroid treatment did display similar results as the conventional or non-treated patients (data not shown), except for the PPARγ protein content in the visceral adipose tissue, which was higher in patients on steroid therapy (*p* = 0.01 compared to patients not on steroid therapy).

### Local production of eicosanoid PPARγ ligands

**Fig. 2:**
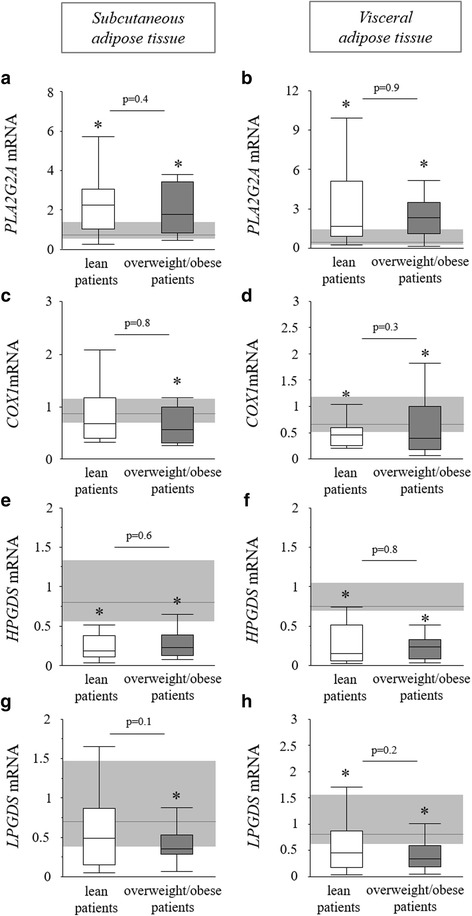
Enzymes involved in prostaglandin PPARγ agonist production in lean and overweight/obese prolonged critically ill patients. Relative mRNA expression of *PLA2G2A* in subcutaneous (**a**) and visceral (**b**) adipose tissue. Relative mRNA expression of *COX1* in subcutaneous (**c**) and visceral (**d**) adipose tissue. Relative mRNA expression of *HPGDS* in subcutaneous (**e**) and visceral (**f**) adipose tissue. Relative mRNA expression of *LPGDS* in subcutaneous (**g**) and visceral (**h**) adipose tissue. Gene expression data are expressed normalized to *RNA18S5* or *GAPDH* and as a fold change of the mean of the healthy controls. *Light gray bar* median and interquartile ranges of healthy controls (*n* = 20), *white* lean prolonged critically ill patients (BMI ≤25 kg/m^2^; *n* = 24), *dark gray* overweight/obese prolonged critically ill patients (BMI >25 kg/m^2^; *n* = 24) [*p ≤ 0.05 compared to healthy controls]

The first step in the production of fatty acid-derived endogenous PPARγ ligands is the mobilization of their precursors arachidonic acid or linoleic acid from phospholipids by phospholipase A2 (PLA2). Gene expression of the major PLA2 subtype *PLA2G2A* was increased in subcutaneous and visceral adipose tissue biopsies of both lean and overweight/obese critically ill patients (Fig. 2a, b). Next, for the production of known PPARγ agonist 15-deoxy-Δ-12,14-prostaglandin J2 (15dPGJ2) [13], arachidonic acid first has to be converted to prostaglandin H2 by cyclooxygenases (COX1 and COX2), followed by synthesis of prostaglandin D2 and subsequent conversion to 15dPGJ2. However, in subcutaneous and visceral adipose tissue biopsies of lean and overweight/obese critically ill patients, gene expression of *COX1* was reduced (Fig. 2c, d) and *COX2* was unaltered (data not shown). Also, the gene expression of the prostaglandin D2 synthases *LPGDS* and *HPGDS* were reduced in subcutaneous and visceral adipose tissue biopsies of lean and overweight/obese critically ill patients (Fig. 2e–h), arguing against an upregulated production of PPARγ-stimulating prostaglandins.

**Fig. 3:**
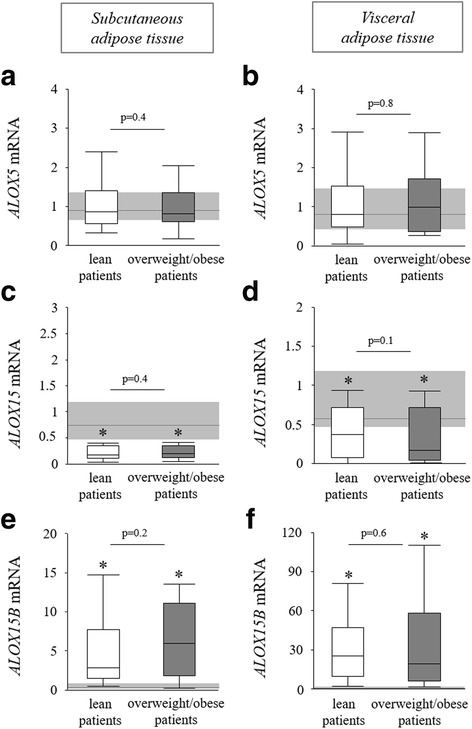
Enzymes involved in lipoxygenase PPARγ agonist production in lean and overweight/obese prolonged critically ill patients. Relative mRNA expression of *ALOX5* in subcutaneous (**a**) and visceral (**b**) adipose tissue. Relative mRNA expression of *ALOX15* in subcutaneous (**c**) and visceral (**d**) adipose tissue. Relative mRNA expression of *ALOX15B* in subcutaneous (**e**) and visceral (**f**) adipose tissue. Gene expression data are expressed normalized to *RNA18S5* or *GAPDH* and as a fold change of the mean of the healthy controls. *Light gray bar* median and interquartile ranges of healthy controls (*n* = 20), *white* lean prolonged critically ill patients (BMI ≤25 kg/m^2^; *n* = 24), *dark gray* overweight/obese prolonged critically ill patients (BMI >25 kg/m^2^; *n* = 24) [*p ≤ 0.05 compared to healthy controls]

**Fig. 4:**
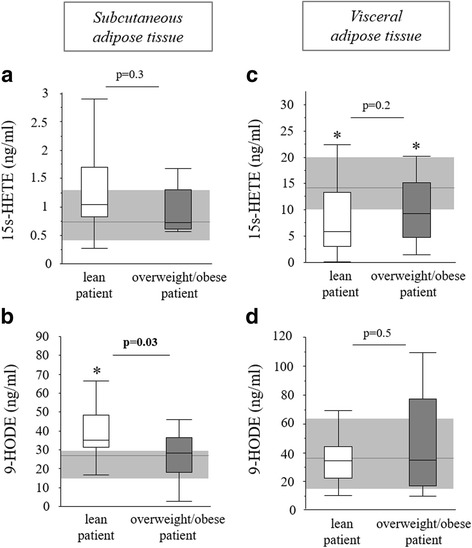
PPARγ ligands in the adipose tissue of lean and overweight/obese prolonged critically ill patients. 15s-HETE concentration in subcutaneous (**a**) and visceral (**c**) adipose tissue. 9-HODE concentration in subcutaneous (**b**) and visceral (**d**) adipose tissue. *Light gray bar* median and interquartile ranges of healthy controls (*n* = 20), *white* lean prolonged critically ill patients (BMI ≤25 kg/m^2^; *n* = 24), *dark gray* overweight/obese prolonged critically ill patients (BMI >25 kg/m^2^; *n* = 24) [*p ≤ 0.05 compared to healthy controls]

Certain lipoxygenase metabolites of arachidonic acid and linoleic acid have also been identified as endogenously produced PPARγ ligands [13]. Within these pathways, gene expression of the key enzyme *ALOX5* was not altered (Fig. 3a, b) and gene expression of the *ALOX15* enzyme was significantly decreased in both subcutaneous and visceral adipose tissue biopsies of lean and overweight/obese critically ill patients (Fig. 3c, d). In contrast, gene expression of the *ALOX15B* enzyme was highly elevated in subcutaneous and visceral adipose tissue of both lean and overweight/obese critically ill patients (Fig. 3e, f). Therefore, we measured adipose tissue concentrations of ALOX5 and ALOX15B end products with PPARγ agonistic activity: the metabolites 15s-HETE and 9-HODE [13]. In subcutaneous adipose tissue, 15s-HETE concentration was comparable in all tested groups (Fig. 4a). However, lean critically ill patients had higher 9-HODE levels than both overweight/obese patients and controls (Fig. 4b). Although the concentration of 15s-HETE was overall much higher in visceral adipose tissue than in subcutaneous adipose tissue, it was reduced in both lean and overweight/obese critically ill patients compared to controls (Fig. 4c). The visceral adipose tissue 9-HODE concentration was not different between groups (Fig. 4d).

None of the measured parameters were differently affected in patients on intensive insulin therapy or on steroid treatment as compared to the conventional or non-treated patients (data not shown).

### In vitro study: adipogenic potential of serum of lean and overweight/obese critically ill patients

**Fig. 5:**
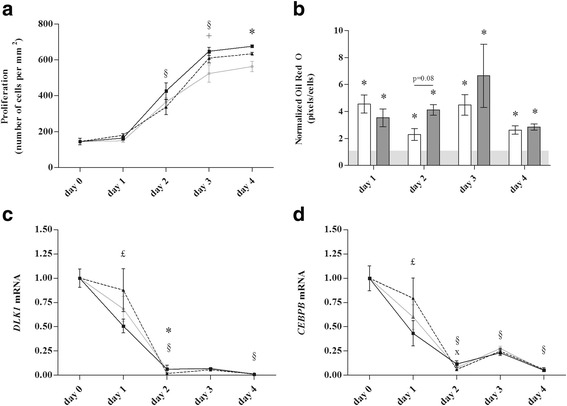
Adipogenic potential of patient serum of lean and overweight/obese prolonged critically ill patients. hADSCs were supplemented with serum from either healthy volunteers (*n* = 47), lean prolonged critically ill patients (BMI ≤25 kg/m^2^, *n* = 20), or overweight/obese prolonged critically ill patients (BMI ≤25 kg/m^2^, *n* = 20). **a** Proliferation presented as the number of cells per square millimeter. **b** Oil Red O staining presented as the number of stained pixels per cells, expressed as a fold of the mean of the healthy conditions per day. **c** Relative mRNA expression of *CEBPB*. **d** Relative mRNA expression of *DLK1.* Gene expression data are expressed normalized to *GAPDH* and as a fold change of the mean of the day 0 condition. *Light gray bar or light gray line* mean of conditions with serum from healthy controls, *white bar or black line* serum from lean patients, *dark gray bar or dotted black line* serum from overweight/obese patients [*p ≤ 0.05 compared to healthy condition, ^§^ *p* ≤ 0.05 compared to previous day, ^+^ *p* = 0.09 compared to healthy condition, ^x^ *p* ≤ 0.05 between lean and overweight/obese conditions, ^£^ *p* ≤ 0.05 between lean and healthy conditions]

As an alternative to locally produced PPARγ ligands, we also investigated whether more upstream signals circulating in the serum of critically ill patients might increase adipogenesis. In an in vitro setup, we therefore studied the adipogenic potential of serum from lean and overweight/obese critically ill patients. Supplementing commercially available hADSCs with serum from either healthy controls or critically ill patients resulted in increased proliferation over time (Fig. 5a). After 3 days, proliferation tended to be higher in patient conditions compared to healthy conditions. On day 4, more cells were observed in patient than in healthy conditions. However, proliferation was not different between conditions with serum from lean or overweight/obese patients (Fig. 5a).

Differentiation to mature adipocytes, measured by cellular lipid accumulation, occurred significantly more in patient compared to control conditions on all tested days (Fig. 5b), but similarly in cells supplemented with either lean or overweight/obese patient serum (Fig. 5b). Also, gene expression of pre-adipocyte marker *DLK1* and adipogenesis-driver *CEBPB* decreased over time in all conditions (Fig. 5c, d). On day 1, this decrease was more pronounced in the lean conditions, after which comparable expression levels were observed in all groups (Fig. 5c, d).

## Discussion

In contrast to our hypothesis, markers of adipogenesis were evenly upregulated in the adipose tissue of lean and overweight/obese prolonged critically ill patients. Furthermore, increased levels of the key adipogenic regulator PPARγ appeared not to be explained by locally produced endogenous eicosanoid agonists. On the other hand, serum from critically ill patients had a clear adipogenic potential, as not only proliferation but especially differentiation of adipose tissue stem cells to mature adipocytes was stimulated in the presence of serum from both lean and overweight/obese prolonged critically ill patients.

We found an increased formation of new, small adipocytes and a clearly upregulated PPARγ expression in subcutaneous and visceral adipose tissue of both lean and of overweight/obese critically ill patients. Overall, lean and overweight/obese prolonged critically patients showed similar signs of adipogenesis. Therefore, the earlier observed difference in muscle wasting between lean and overweight/obese prolonged critically ill patients and rodents [6] is most likely not associated with an altered adipogenic response. The current observation of an increased amount of small adipocytes in both lean and overweight/obese prolonged critically ill patients might suggest that rather than having excess adipocytes, it is the excess energy that is available in the adipose tissue of overweight/obese patients which might play a beneficial role in preventing critical illness-induced muscle wasting. This is in concordance with the previously observed skeletal muscle protection in obese critically ill mice who displayed an increased loss of adipose tissue mass [6].

PPARγ not only is the master regulator of adipogenesis but also plays an important anti-inflammatory role in macrophages [13, 14]. Despite the abundant in vitro documentation that specific fatty acid metabolites are potent PPARγ ligands, only recently physiologically relevant endogenous PPARγ ligands have been identified. Cell-specific increased expression of metabolizing enzymes can lead to the production of specific prostaglandins and lipoxygenase metabolites [10, 15–17]. Especially hematopoietic cells are thought to be the local producers of these endogenous PPARγ ligands [13, 17]. We previously documented an increased accumulation of alternatively activated M2 macrophages in adipose tissue of critically ill patients [5, 7]. Activation of PPARγ by fatty acids could drive this macrophage M2 polarization during critical illness [18]. Such alternatively activated M2 macrophages are able to produce endogenous fatty acid-derived eicosanoid PPARγ ligands that can stimulate adipogenesis [10]. Furthermore, in response to inflammatory stimuli such as zymosan peritonitis or carrageenan-induced pleuritis, the endogenous hematopoietic levels of the prostaglandin 15dPGJ2 have been shown to increase in vivo [16, 19]. Cytokines such as interleukin (IL)-4 can stimulate the production of the endogenous PPARγ ligands 13-HODE, 9-HODE, and 15s-HETE through upregulation of specific lipoxygenases [13, 17, 20]. However, again in contrast to what we expected, gene expression of the enzymes involved in the production of 15dPGJ2 were decreased rather than increased in adipose tissue biopsies of prolonged critically ill patients. Also, *ALOX15*, involved in the production of 13-HODE, was downregulated. Although *ALOX15B* was strongly increased, its end product 15s-HETE was not elevated in subcutaneous adipose tissue and even decreased in visceral adipose tissue biopsies. Only in subcutaneous adipose tissue of lean patients, we found a significantly higher concentration of the PPARγ ligand 9-HODE. Overall, our data argue against the local production of known eicosanoid PPARγ agonists, at least in the prolonged phase of critical illness.

As an alternative to locally produced PPARγ ligands, we also investigated whether more upstream signals circulating in the serum of critically ill patients might increase adipogenesis. We observed that not only proliferation but especially differentiation of adipose tissue stem cells was stimulated in the presence of serum from both prolonged lean and overweight/obese patients. The identification of which serum component(s) are responsible for the stimulation of proliferation and differentiation was beyond the scope of this study.

Insulin therapy did not affect the studied markers of adipogenesis, as was observed in earlier studies [4, 5]. A small effect of steroid treatment was observed in the postmortem study, but steroid treatment was an exclusion criterion for the in vitro study.

Why adipogenesis is increased during critical illness cannot be concluded from this study. We speculated earlier that these newly formed adipocytes, which are more lipid and glucose storage apt than older lipid-loaded adipocytes, might improve the metabolic profile of the patient and reduce potential detrimental effects of high circulating levels of these metabolites [21]. However, the observation that obesity prevents critical illness-induced muscle wasting, but appears to increase the use of adipose tissue lipid stores, argues against this hypothesis [6]. Maybe, adipogenesis is merely a side effect of the profound endocrine alterations observed during critical illness.

The study has important limitations. First, we studied postmortem adipose tissue biopsies from critically ill patients. Although biopsies were harvested minutes after death, we cannot exclude the interference of agonal hypoxia with our results. However, such interference would have similarly affected samples from lean and overweight/obese patients. Furthermore, increased adipogenesis was also observed in in vivo adipose tissue biopsies from critically ill patients [5, 7]. Secondly, since we mainly studied overweight to mildly obese patients, possibly severely to morbidly obese patients might display a different adipogenic response. However, the findings on the protection against muscle wasting and weakness were also observed in an overweight/obese patient population with a similar BMI range as the current study [6]. Thirdly, although patients were matched for the presence of diabetes, information on the type of diabetes and on the use of antidiabetic drugs, such as thiazolidinediones, prior to ICU stay, is missing, both of which might have affected adipogenesis in these patients. Lastly, our in vitro study was designed to mimic the complex and multifactorial condition of critical illness. Although this in vitro setup enables us to get a global overview of the adipogenic response during critical illness, it cannot distinguish between specific effects of individual factors present in the serum of critically ill patients.

## Conclusions

Against what we hypothesized, adipogenesis was increased independently of initial BMI in prolonged critically ill patients. Endogenous adipose tissue production of PPARγ agonists was not observed in these patients. However, serum of prolonged critically ill patients was a strong stimulator of proliferation and especially differentiation of adipose tissue stem cells. The latter findings suggest a humoral rather than a paracrine adipogenic signal during prolonged critical illness. Importantly, rather than having excess adipocytes, it might be the excess energy that is available in the adipose tissue of overweight/obese patients which might be imperative during critical illness. Of interest, if the usage of stored substrates from the adipose tissue as a source of energy indeed can protect the muscle during critical illness, this raises the question whether supplementing lean patients with lipids could mimic such muscle-sparing effect.

## Abbreviations

15dPGJ2: 15-Deoxy-Δ-12,14-prostaglandin J2
15s-HETE: 15-Hydroxyeicosatetraenoic acid
9-HODE: 9-Hydroxyoctadecadienoic acid
A/A: Antibiotic-Antimycotic
ALOX15: Arachidonate 15-lipoxygenase
ALOX15B: Arachidonate 15-lipoxygenase type B
ALOX5: Arachidonate 5-lipoxygenase
ANOVA: Analysis of variance
BMI: Body mass index
CEBPB: CCAAT/enhancer-binding protein beta
COX1: Cyclooxygenase 1
COX2: Cyclooxygenase 2
DLK1: Delta-like 1 homolog
FBS: Fetal bovine serum
GAPDH: Glyceraldehyde-3-phosphate dehydrogenase
hADSCs: Human adipose-derived stem cells
HPGDS: Hematopoietic prostaglandin D synthase
ICU: Intensive care unit
IL: Interleukin
LPGDS: Prostaglandin D2 synthase
PLA2: Phospholipase A2
PLA2G2A: Phospholipase A2 group IIA
PPARγ: Peroxisome proliferator-activated receptor gamma
RNA18S5: RNA, 18S ribosomal 5
RPMI: Roswell Park Memorial Institute
SEM: Standard error of the mean
